# Sex differences in COVID-19 mortality: A large US-based cohort study (2020–2022)

**DOI:** 10.3934/publichealth.2024045

**Published:** 2024-07-09

**Authors:** Samer A Kharroubi, Marwa Diab-El-Harake

**Affiliations:** 1 Department of Nutrition and Food Sciences, Faculty of Agricultural and Food Sciences, American University of Beirut, P.O.BOX: 11-0236, Riad El Solh 1107-2020 Beirut, Lebanon; 2 School of Health and Related Research, The University of Sheffield, Regent Court, 30 Regent Street, S1 4DA, Sheffield, UK

**Keywords:** sex differences, COVID-19, mortality, Healthjump data, US cohort

## Abstract

**Background:**

In the present study, we aim to assess the trend in mortality in COVID-19 by time and sex in a large cohort using Datavant's Death Index database. The main objectives of this study are to analyze mortality cases over time, which are categorized by sex and age, and to identify potential reasons for the observed differences.

**Methods:**

This is a retrospective cohort containing information on deceased individuals in the United States and Canada (n = 4,384,265). We included adult male and female patients with a clinical diagnosis of COVID-19 (January–December 2022) (ICD-10 code: U07.1). Mortality cases for males and females were presented over a three-year period of COVID-19 pandemic. Sex ratios presenting the change of mortality cases over time was also computed as the number of diagnosed males over female patients. Sex-differences in the mortality rates were illustrated by age groups.

**Results:**

In 2020, mortality cases increased to reach up to 200,000 cases per day and fluctuated due to social and/or cultural events in the US. In 2021, mortality cases reached the highest peak over the time period despite the US vaccine rollout due to holiday gatherings during November and December 2021, as well as the spread of a more contagious strain of the virus. In 2022, mortality cases decreased due to widespread vaccinations and a rise in natural immunity following the first Omicron surge. Furthermore, the proportion of COVID-19 cases in males and females remained stable during the pandemic; however, the number of diagnosed male patients markedly increased during the first months of 2022. Gender discrepancies suggest the role of various factors such as occupation, underlying comorbidities, and behavioral and immunological factors.

**Conclusion:**

Our study highlights higher mortality rates observed among males, suggesting that several factors may contribute to such differences, including social, behavioral, and biological factors. Our findings highlight the importance of implementing sex-specific treatment approaches in COVID-19 patients.

## 1. Introduction

Understanding the factors that increase the risk of disease, death, and health disparities requires the collection of data that is sex-disaggregated. Recent studies suggest that men diagnosed with the 2019 new coronavirus disease (COVID-19) had a more severe illness and a greater fatality rate than women [1]–[3]. For instance, the majority of the 84 nations analyzed for COVID-19 mortality by sex showed greater case fatality ratios (CFR) in males, with the Netherlands having one of the uppermost CFRs for men to women within the first year [4]. Older age appears to be the biggest risk factor for dying from COVID-19. Additionally, some co-existing conditions, primarily obesity, cardiovascular disease, hypertension, and diabetes, have been linked to serious illness outcomes [5],[6]. Results point to a varied impact of COVID-19 on outcomes in males compared to women; however, more research is needed.

It is unclear if these sex differences are related to men's generally worse health and shorter life span as compared to women their age, or if men have a specific COVID-19 disadvantage. It is crucial to perform a sex-disaggregated analysis of COVID-19 because it facilitates risk stratification that enables sex-specific management and treatments for patients. In this study, we present a detailed description outlining the disparities in COVID-19 mortality between males and females during the first and second wave of the COVID-19 pandemic in the United States to gain a further understanding of temporal trends in these discrepancies. Our aim is to explore the sex-differences in COVID-19 using a large US-based cohort. More specifically, the objectives are as follows: 1) to assess the trend in mortality rates of COVID-19 patients by time and sex in a large US-based cohort; 2) to study the sex-ratio variation in mortality cases over time; and 3) to determine sex-differences in mortality by age groups. Sex-disaggregated research is important to understand the risk factors of poor outcomes and health inequities, as well as to guide COVID-19 management.

## 2. Materials and methods

### 2.1. Study design and data source

This retrospective cohort study aims to assess the mortality trend associated with COVID-19 across time and gender within a large cohort based in the United States and Canada. The study population includes data on the mortality rates of male and female patients with a clinical diagnosis of COVID-19 from January 1, 2020, to December 31, 2022 (ICD-10 code: U07.1.), which are accessible through the COVID-19 Research Database consortium (https://covid19researchdatabase.org).

The COVID-19 Research Database was initiated following approval from institutional review boards, patient advocacy groups, and ethics committees (https://covid19researchdatabase.org/). Patient consent was exempted due to the use of Health Insurance Portability and Accountability Act (HIPAA) de-identified data, HIPAA limited data, or non-HIPAA covered data, supported by strong governance protocols regulating data access. This exemption includes all research conducted within the COVID-19 Research Database. For further information on ethics and research intent, please refer to the COVID-19 Research Database Statement of Ethics and Intent available at https://covid19researchdatabase.org/statement-of-ethics/.

### 2.2. The mortality database

Datavant, which is a company that enhances the Social Security Administration death master files by incorporating information from newspapers, funeral homes, and memorials to establish a comprehensive database at the individual level, encompassing over 80% of annual deaths in the United States, provided detailed mortality statistics between 2020 to 2022. We extracted and analyzed the mortality data for patients with available electronic health records (EHR) from 2020 to 2022. Then, among these, we retrieved the demographic information and outcomes for approximately 4 million patients with a test-confirmed diagnosis of COVID-19.

Datavant's Death Index includes information on deceased individuals in the United States and Canada. The data sources of the death index dataset include the government, private obituaries, and private claims sources. Datavant's Death Index combines death records sourced from the NTIS Social Security Administration's Death Master File (referred to as “SSA”), as well as third party data from public and private obituaries (referred to as “Obit” data) from 2010 onward. About 99.6% of the data pertains to deaths in the United States, and the rest are from Canada. For each record, the data indicate the date of death, the date of birth, and the gender of the deceased.

### 2.3. Data analysis

Mortality cases for males and females were presented over a three-year period during the COVID-19 pandemic from 2020 to 2022. We analyzed sex-based differences in mortality cases with a clinical diagnosis of COVID-19. Sex ratios presenting the change of mortality cases over time was also computed as the number of diagnosed male patients over female patients. Sex-differences in the mortality rates were illustrated by age groups.

## 3. Results

**Figure 1.**
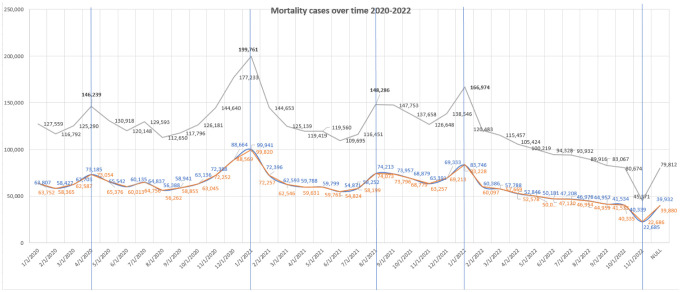
Mortality cases over time 2020–2022 using a large, US-based Cohort (n = 4,384,265). Blue lines refer to mortality data for males and orange lines refer to mortality data for females.

Figure 1 shows the mortality cases between 2020 and 2022. In 2020, the death cases increased up to 200,000 cases using a large US-based Cohort study. In 2021, the death cases decreased compared to the previous year and fluctuated between 200,000 cases and 160,000 cases. In 2022, the mortality cases abruptly decreased from 160,000 cases to less than 50,000 cases.

More specifically, the COVID-19 mortality cases fluctuated between 2020 and 2022. COVID-19 cases and deaths initially peaked in April and May 2020 (146,239 and 130,918 respectively); however, after a brief reduction in the June deaths (120,148 cases), the death cases began to rise again, reached a second peak during July 2020 (129,593 cases), declined in August (112,650), and returned to climb again from September (117,796) to October (126,181). The lowest number of deaths occurred in February (116,792), August (112,650), and September (117,796). Furthermore, the number of COVID-19 cases increased suddenly to reach 144,640 in November 2020. The number of cases gradually increased to reach a third peak on December 1, 2020.

In 2021, the number of death cases reached its highest record over the period 2020–2022, reaching 199,761 cases on January 1, 2021 (Winter 2021, wave 2). The frequency of deaths steeply decreased afterwards during Spring–Summer 2021 to reach 109,695 cases in June 2021. After that, the number of cases slightly increased to reach the second peak with 148,658 cases in August 2021 (Summer 2021). Moreover, the death cases remained stable during August and September 2021. This was followed by a sharp decrease in deaths to reach 126,648 cases in November 2021. Finally, the number of death cases suddenly increased from November to December 2021 to reach 138,546 cases.

Alternatively, in 2022, the number of deaths increased between December 2021 and January 2022, reaching 166,974 cases. There was modest decline in the mortality rates in February 2022 to 120,483, and somehow remained stable and controlled until October. Then, the frequency of COVID-19 deaths significantly decreased, reaching the lowest death record with 45,371 deaths in October 30. However, in November 2022, the mortality rates increased again to the lowest record over the years (79,812 in December 2022).

**Figure 2.**
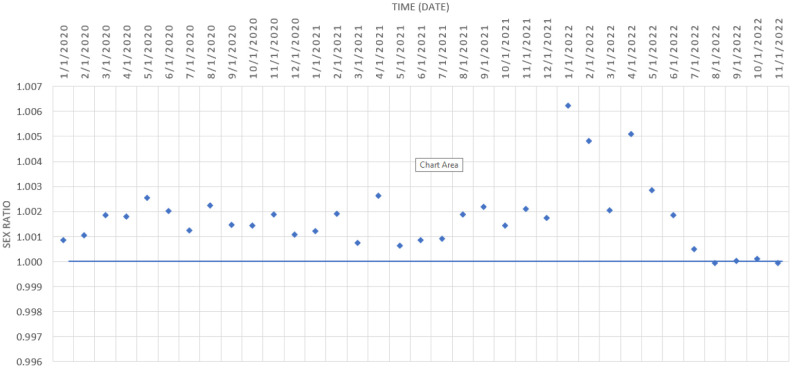
Sex Ratios of mortality cases over time within study period, calculated as the number of diagnosed male deaths over female deaths.

In terms of sex disparities in COVID-19 deaths over time, no significant differences were observed between men and women by pandemic month in our study (p = 0.06) (Figure 1). Figure 2 shows the sex ratios of the mortality cases over time. The proportion of COVID-19 cases in males and females remained stable during the pandemic (Figure 2); however, the number of diagnosed male patients markedly increased during January (1.006), February (1.0048), and April 2022 (1.005). Then, the number of diagnosed females was equal to the number of diagnosed males by the end of 2022.

**Figure 3.**
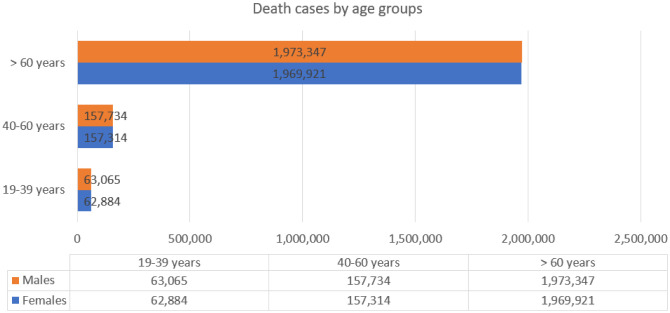
Death cases by age groups.

Results from our cohort showed that the number of death cases increased by the age group (Figure 3). Older adults had the highest number of deaths during 2020–2022. For instance, a higher number of deaths existed among adults aged 40 to 60 years (315,048) compared to that of adults aged 19–39 years (125,949). COVID-19 death rates were higher for men compared to women for all age groups: 19–39 (63,065 females, 62,884 males), 40–60 (157,734 males, 157,314 females), and 60 and over (1,973,347 males, 1,969,921 females). No sex-dependent statistical differences were observed for the number of COVID-19 deaths across age groups (p = 0.952).

To finally emphasize the significance of our study, Table 1 compiles and synthesizes findings from other studies across various areas.

**Table 1.** Summary table for other studies.

**Authors/date**	**Aim of study/paper**	**Type of study/information**	**Main findings/conclusions**
Islam N, Jdanov DA (2023) [7]	To examine how mortality estimates from England and Wales, Spain, and Italy were influenced by population age and sex	Cross-sectional study	• The greatest predictor of death is age, which is also linked to multimorbidity, which affects mortality in and of itself. • Another important element is sex. Because women typically live longer than men do in most nations, women are overrepresented in older age groups. For instance, the ratio of women to men aged 65 or over was 54:46 in England and Wales in 2020. • Women in Spain, not Italy, had the lowest age-standardized mortality, whereas English women continued to have the worst.
Danielsen AC, Lee KM, Boulicault M, et al. (2022) [8]	To study sex disparities in COVID-19 cases and mortalities across U.S. states, derived from the unique 13-month dataset of the U.S. Gender/Sex COVID-19 Data Tracker.	Longitudinal study	• The findings show that the gender gap in COVID-19 cases and deaths varies significantly over time and between states. • According to these statistics, there appears to be a small sex discrepancy when it does exist, and it probably fluctuates depending on context-sensitive factors including occupation, race/ethnicity, health practices, and other indicators of social experience.
Grande E, Fedeli U, Pappagallo M, et al. (2022) [9]	To investigate cause-specific mortality in Italy from March 2020 to April 2020 and the variation in mortality rates compared with those in 2015–2019 regarding sex, age, and epidemic area.	Cross-sectional study	• Results reveal impact of Covid-19 pandemic on cause-specific mortality from March 2020 to April 2020, including age, gender, and territorial differences. An excess of deaths for influenza and pneumonia in the months of March and April was observed in the US. Air pollution might explain the observed geographical pattern of the increase in mortality for COVID-19 in Italy. In the younger age groups, especially in males, there was an excess in mortality from pneumonia and other respiratory diseases, suggesting an underestimation of COVID-19 cases.
Kharroubi SA, Diab-El-Harake M (2022) [10]	To investigate potential sex differences in lifestyle factors and clinical comorbidities among diagnosed patients, as well as the socioeconomic features of COVID-19 male and female patients.	Retrospective cohort study	• Significant sex-dependent differences were found in the COVID-19 risk variables and illness comorbidities using a sizable US population. After controlling for important factors including age, education, and ethnicity, male patients in particular had a high frequency of underlying comorbidities, such as abnormal clinical and lab results, hypertensive disorders, and diabetes. • This study suggests that comprehending the disparities in patient outcomes across male and female cohorts would facilitate the development of a gender equity responsive strategy for the COVID-19 pandemic and augment the efficacy of medical practice, legislation, and interventions.
Yoshida Y, Chu S, Fox S, et al. (2022) [11]	To examine the association of comorbidities, inflammatory biomarkers, and severe outcomes in men and women hospitalized for COVID-19.	A retrospective cohort analysis	• Inpatient mortality varies by gender and is correlated with comorbidities and biomarkers. Among patients hospitalized with COVID-19, the four most common fatal comorbidities were moderate to severe liver disease, renal disease, metastatic solid tumor, and myocardial infarction. These four coexisting conditions continued to be the most deadly in both sexes, with women at a larger risk than males. Procalcitonin and NT proBNP were the best predictors of mortality, with abnormal increases of CRP, ferritin, procalcitonin, NT proBNP, neutrophil and platelet counts, and lymphocytopenia strongly linked to the risk of death.
Péterfi A, Mészáros Á, Szarvas Z, et al. (2022) [12]	A systematic search on PubMed for articles published between 2019 and 2021 to identify any cohort and case-control studies that investigated the relationship of comorbidities and COVID-19 mortality among the elderly, defined as 60 years of age and above.	Systematic review	• According to the included studies, there is a correlation between higher mortality rates in populations of elderly and the prevalence of specific disorders as diabetes, cancer, respiratory, kidney, cardiovascular, and nervous system diseases. Possible mechanisms include SARS-CoV-2 virus attacks endothelial cells, causing damage to tiny arteries and encouraging microvascular dysfunction, inflammation, and thrombosis. As a condition worsens, the link between comorbidities and COVID-19 mortality in the elderly may alter, with stronger observed effect sizes as the disease advances.
Dorrucci M, Minelli G, Boros S, et al. (2021) [13]	To compare all-cause excess mortality between the two waves that occurred during the year 2020 using nationwide data.	Cross-sectional study	• Similar to other European countries, the COVID-19 pandemic scenario in Italy can be summed up in three phases: a first wave that began at the end of February and ended at the end of May, marked by a sharp increase in cases and deaths as well as a high territorial concentration, primarily in the north of the country; a transitional phase that began in June and ended in mid-September, with low viral diffusion; and a second wave that began at the end of September 2020 and continued until the first half of November before the cases again declined. Men presented a higher mortality risk than women during the second wave, even though not significantly. The highest contribution to excess mortality during the whole period covered by this study was observed among people aged 80 years and older, with a further increase during the second wave.
Mahmoud M, Carmisciano L, Tagliafico L, et al. (2021) [14]	To identify the chronic clinical conditions and the comorbidity clusters associated with in-hospital mortality in a cohort of older COVID-19 patients who were admitted to the IRCCS Policlinico San Martino Hospital, Genoa, Italy, between January and April 2020.	This was a retrospective cohort study including 219 consecutive patients aged 70 years or older and is part of the GECOVID-19 study group.	• According to the findings, individuals with dementia and cerebrovascular illness had higher mortality rates connected to COVID-19, and these disorders continued to have an independent relationship with all-cause in-hospital mortality. Specifically, this may be due to a higher likelihood of hospitalization, the requirement for intensive care, and mortality when heart failure, metabolic disorders, stroke, and dementia among patients with these comorbidities. In addition, the inflammatory response may be involved in the worsened clinical course of COVID-19 in older and frail patients.
Chang WH (2020) [15]	To understand whether women belong to a high-risk group or a group with weak resistance.	Review article	• Women outperform men in terms of illness incidence and fatality. The primary cause is that women's immune systems are stronger than men's, in addition to their own hormonal defenses. In addition, women observe safety precautions more strictly and engage in more protective behavior (such as hand washing), which significantly lowers the risk of infection.
Griffith DM, Sharma G, Holliday CS, et al. (2020) [16]	To understand the disproportionate death rate among men using a biopsychosocial approach.	Commentary	• Biological factors: Women often develop higher innate and adaptive immunological responses than men because the X chromosome has a high number of immune-related genes. Sex hormones including progesterone, estrogen, and androgens, as well as sex chromosomal genes, contribute to this unequal control of immunological responses in men and women. • Psychosocial and behavioral factors: Men are more likely than women to participate in high-risk activities that increase their chance of getting COVID-19 including higher rates of tobacco use and alcohol consumption. For example, men are less likely than women to have stated that they have been avoiding intimate physical contact with people or big public gatherings. Additionally, men are less likely than women to efficiently seek medical assistance, wear masks, and wash their hands. • Other factors include age and geography. For example, a US study showed that although older men perceived their risks of COVID-19 to be higher than those of younger men, older men made the fewest behavior changes across age and gender groups. • There are variations in occupational risk for men and women. In the United States, a larger number of women are employed as social and healthcare workers. However, men are more likely to be low-skilled or low-paid occupations (e.g., food processing, transportation, delivery, warehousing), which are associated with a greater risk of mortality.
Scortichini M, Schneider dos Santos R, De'Donato F, et al. (2020) [17]	To analyze the excess mortality across the 107 Italian provinces, stratified by sex, age group and period of the outbreak.	Cross-sectional study	• The impact was slightly higher in men compared with women. The risk varied by age, with the highest excess mortality in the group of people aged 70–79 years old and with a lower but measurable risk evident in those aged <60. The analysis by week reveals varied patterns, with the risk restricted to a shorter time frame (March to mid-April) in Central and Southern Italy, and more delayed affects in women and the elderly. These variations are probably caused by the lockdown rules were implemented and by the contributions of differential risk pathways, which include deaths directly linked to COVID-19 and indirect deaths from other causes including disturbances in the healthcare system.
Raparelli V, Palmieri L, Canevelli M. (2020) [18]	A sex-stratified analysis to examine variations in the clinical course and presentation between male and female COVID-19 fatality cases in Italy.	Cross-sectional study	• In the current analysis, men and women experienced distinct clinical symptoms and care transitions. Compared to women, men's COVID-19-related mortality was associated with different clinical symptoms and treatment transitions. Even though this elderly cohort had a high overall burden of multimorbidity, males were more likely than women to have IHD, COPD, and CKD, whereas women were more likely to have dementia and autoimmune disorders. Men were more likely than women to experience the clinical symptoms of COVID-19 death, such as fever as a sign of start and the incidence of AKI.
Sanyaolu A, Okorie C, Marinkovic A, et al. (2020) [19]	To examine the comorbid conditions, the progression of the disease, and mortality rates in patients of all ages, infected with the ongoing COVID-19 disease.	Electronic literature review search	• Patients with COVID-19 disease who have comorbidities, such as hypertension or diabetes mellitus, are more likely to develop a more severe course and progression of the disease. • Older patients, especially those 65 years old and above who have comorbidities and are infected, have an increased admission rate into the intensive care unit (ICU) and mortality from the COVID-19 disease.

## 4. Discussion

The present study was the first to describe mortality cases over time and to examine reasons for sex- and age-differences in mortality rates among COVID-19 patients. From this large cohort analysis, we confirmed a significant role of sex in COVID-19 mortality. The risk of death may be explained by several factors such as the number of comorbidities, occupation, behavioral factors, etc. These results enable us to identify sex-specific risk factors related to COVID-19. A sex-disaggregated analysis is essential because it allows for risk classification, which allows for sex-specific patient management and treatment.

In the present paper, we assessed the trend in COVID-19 mortality rates by time and sex. In 2020, the mortality cases increased to reach up to 200,000 cases per day and fluctuated due to national health restrictions and social or cultural events in the US. More specifically, on March 11, 2020, the World Health Organization (WHO) declared that COVID-19 could be classified as a pandemic due to its concerning levels of transmission and its severity [20]. Shortly thereafter, many states initiated shutdown measures. However, by mid-April, a significant number of states were documenting widespread instances of COVID-19, corresponding to the spike in deaths during the Spring season and the easing of restrictions. On April 13, 2020, countries considered easing restrictions, while others contemplated the implementation of similar measures, particularly those in Africa, Asia, and Latin America, which have lower to middle-income economies. However, in nations with significant impoverished populations such as the United States, the feasibility of adopting stay-at-home orders and other restrictions, as observed in some wealthier nations, might be limited [21]. Later, the months of August and September of 2020 had the fewest fatalities. These were summer months, where there may have been reduced virus exposure due to less virus dissemination. During the summer months in the United States, cities and states either slowed down their reopening plans or reimposed restrictions. Furthermore, the number of cases gradually increased to reach the third peak on December 1st, 2020, which corresponded with Winter break and Christmas holidays in the United States, whereby the number of COVID-19 cases reached 177,233. During the last two months in 2020, the number of death cases increased as people engaged in risky Christmas and New Year holiday gatherings in late 2020.

In 2021, the mortality cases reached the highest peak over the time period (2020–2022), despite the US vaccine rollout due to holiday gatherings [22] during November and December 2021, as well as the spread of a more contagious strain of the virus in the United States and a slower-than-expected rollout of vaccines [23]. Furthermore, deaths were higher due to a delay in the vaccine release due to the spread of a new strain of the virus. Then, President-elect Joe Biden postponed the vaccine's introduction since many experts prioritized the Pfizer/BioNTech and Moderna vaccines, since they were estimated to be roughly 95% effective [23].

The death cases decreased during mid-2021 as the vaccination rates increased with a shortage among younger adults and residents of the United States. As vaccination coverage improved due to health and governmental plans and the implementation of compensatory actions, the mortality rate decreased until the end of 2021. Additionally, COVID-19 fell from the first to the seventh greatest cause of mortality in the US due to a sharp increase in the immunization rates. However, in the summer of 2021, cases abruptly increased again. This may have occurred due to low vaccination rates, notably among younger adults and residents of some states; moreover, the federal government fell short of its July 4 deadline for getting approximately 70% of adults vaccinated. Afterwards, the number of deaths were stable for August and September 2021 due to vaccination approval. On August 18, 2021, the Center for Disease Control (CDC) declared plans for Covid-19 vaccine booster shots. The Food and Drug Administration (FDA) fully approved the Pfizer-BioNTech COVID-19 vaccine for all people ages 18+ on August 23, 2021 [21]. As of August 25, 2021, approximately 73% of adults (aged 18 and above) in the US received at least one dose of the COVID-19 vaccine.

The number of deaths gradually decreased in fall 2021 (September–November). Additionally, the drop and mitigation for the COVID-19 cases and deaths during fall 2021 could be related to the increased CDC funding for COVID-19 prevention and control, as well as strengthening the health authorities' recommendations for full vaccination. On September 17, 2021, the Biden Administration invested $2.1 billion through the CDC to provide state, local, and territorial public health departments with the funding they required to fight infectious diseases such as COVID-19, improve laboratory capacities, and prevent infections in healthcare settings. Furthermore, in November 2021, all non-citizens traveling to the U.S. were mandated to be fully vaccinated and provide evidence of their vaccination status in order to board flights bound for the US [24]. In addition, on November 19, 2021, and in accordance to the CDC's robust recommendations, everyone who received a COVID-19 vaccine from either Johnson & Johnson, Pfizer-BioNTech, or Moderna and was 18 years of age or older should were recommended to obtain a booster shot when they were fully immunized. Nevertheless, the number of deaths increased back again to a higher death record in December 2021. This increase may be explained by the more infectious COVID-19 Delta variant, insufficient vaccination rates, and higher COVID-19 cases, hospitalizations, and deaths [25]. On December 1, 2021, the California and San Francisco Departments of Public Health identified the initial instance of the Omicron variant in the US. According to statistics made public by the CDC, the Omicron variant was roughly 1.6 times more contagious than the Delta variant [24].

In 2022, the mortality cases were stable and decreased due to widespread vaccinations, a rise in natural immunity following the first Omicron surges, and the availability of anti-COVID therapeutic drugs. In the first half of 2022, there was a notable surge in fatalities following a rise in hospitalizations and admissions to intensive care units, driven by the highly transmissible Omicron variant of the virus. Though the surge of the Omicron cases decreased across the country in 2022, hospitalizations and deaths were expected to remain high throughout the month. This was mainly due to a lag in COVID-19 deaths based on new infections and hospitalizations by a few weeks, which explained why fatalities continued to increase even as the number of cases declined, in addition to the critical gaps in the vaccination coverage in the United States [26]. The United States reported approximately 1 million new COVID-19 infections on January 3, 2022, which was the largest daily amount of any nation in the globe. In just one week, the number of COVID-19 patients in hospitals increased by about 50%.

In November 2022, the mortality rates increased again to 79,812 in December 2022. The rise in deaths in the last months of 2022 was primarily due to holiday gatherings. Other concerns included the decreased vaccine immunity and relatively poor booster uptake compared to first wave of vaccinations. Data from 2022 indicates a notable decrease in COVID-19 mortality during the third year of the pandemic compared to the previous two years (p < 0.05). Preliminary figures from Johns Hopkins University revealed over 350,000 COVID-19 fatalities in 2020, surpassing 475,000 in 2021. However, in 2022, the toll decreased to over 267,000 deaths. Despite this decline, COVID-19 remained the third leading cause of death in the United States for the third consecutive year. It is important to note that over the course of the pandemic, the quality of reporting of COVID-19 deaths by healthcare workers and medical professionals improved [27].

Regarding sex-based disparities in COVID-19 mortality across time, our study did not find any significant differences between men and women. However, analyzing gender discrepancies in COVID-19 mortality trends within the United States suggests the roles of various factors. First, mortality patterns exhibited significant temporal and regional variability. Second, social and behavioral factors such as occupation, behavioral habits, and underlying health issues likely contributed to sex-based disparities in COVID-19 mortality [27] (Table 1). For instance, men are more likely to work in industries such as transportation, manufacturing, meatpacking, agriculture, and construction, which have higher exposure and fatality rates to the COVID-19 virus. Men are more likely to endure homelessness and incarceration compared to women, both of which increase their chance of contracting a virus. Women are more likely to report washing their hands, wearing masks, and following social isolation rules compared to men, all of which may reduce their chance of catching the infection. Moreover, women are more likely to receive vaccinations [15],[16],[28]. Similar to this study, a data tracker for COVID-19 cases and deaths in the US revealed that there were no significant disparities in the infection rates between men and women. However, when examining the death rates, which consider the number of deaths among either men or women relative to the total population of each gender, it was frequently observed that men had higher mortality rates compared to women. On the other hand, other studies attributed sex differences to genes, hormones, and immune responses [29]. Similarly, further evidence from Italy found different mortality rates in time depending from external factors such as accidental falls [9], a person's capacity to escape immunity [30], and a different pattern of the Sars-Cov2 diffusion detected during the second wave, both in terms of quantitative and the geographical pattern [13],[17]. Some studies suggest that elevated COVID-19 mortality rates in men may be linked to a heavier burden of comorbidities [21],[22]. However, other research showed that a greater mortality in women was associated with a higher frequency of comorbidities such as moderate/severe liver disease, dementia, metastatic solid tumor, and heart failure [11],[18]. Sex is a critical factor in mortality. In most countries, women tend to have a longer life expectancy compared to men, this leading to a higher proportion of women in older age brackets. In England and Wales in 2020, the ratio of women to men aged 65 or older was 54:46. Moreover, as age increases, male mortality rates consistently exceed female mortality rates. Therefore, age standardization affects the mortality estimates differently for men and women.

The findings of the current study should be interpreted considering the study's limitations. First, the lack of data on confounding factors, such as ethnicity and a patient's co-morbidities and severity [31], is one of the risk factors for COVID-19 mortality. Our study only included a small amount of sociodemographic information, health care access/use, and clinical information due to the unavailability of the data; as a result, our analysis is susceptible to residual confounding. The only available outcome was all-cause mortality. Furthermore, as our study was observational, causal inferences cannot be drawn. In addition, mortality among patients was assumed to be linked to COVID-19 rather than comorbidities from other diseases. Finally, electronic health record (EHR) systems may be susceptible to potential biases in data recording, which could stem from differences in the system functionalities, layout, coding systems, user knowledge, education regarding EHR utilization, and variations in the data extraction tools and processing methods [26]. Despite these restrictions, the extensive EHR data provided enough strength for a sex-stratified study and brought attention to the notable disparities between the sexes in COVID-19 death rates in the United States. Our results highlight the significance of applying COVID-19-specific sex-specific prevention and therapeutic approaches.

Our cohort's findings revealed that the number of mortality cases increased with age with no statistically significant difference. Even though COVID-19 can impact individuals of all ages, older adults were disproportionately affected during the initial year of the pandemic, as indicated by the NCHS data: 81% of the COVID-19 fatalities in 2020 (totaling 282,836) were individuals aged 65 and older. Following heart disease and cancer, COVID-19 was ranked as the third leading cause of death within this age group [27]. Several factors contribute to the heightened vulnerability of older individuals to COVID-19 mortality. The concept of a “pre-existing mortality gap” may help explain the sex disparity observed in COVID-19 fatalities, rather than a specific vulnerability among males to the virus. Prior to the emergence of COVID-19, men exhibited a shorter life expectancy [32], potentially influenced by higher occurrences of specific chronic conditions [33], engagement in riskier behaviors [34], and being employed in more hazardous occupations [35]. In addition, one probable description of the observed increased mortality in older patients could be patient comorbidities [12],[14],[19]. Conditions such as hypertension and obesity are correlated with increased mortality rates from COVID-19 [7]. Furthermore, changes associated with immune senescence might explain the increased vulnerability among older age patients. The immune response to vaccines is influenced by age-related changes within the immune system, biochemical alterations brought on by aging, which are connected to an increased vulnerability to infectious infections, increased inflammatory phenotypes that cause immune dysfunction, age-related cell changes and soluble mediators of both the innate and adaptive immune responses within lymphoid and non-lymphoid peripheral tissues, and a low-grade, sterile, persistent inflammation known as “inflammaging” [36],[37]. Conditions such as hypertension, peripheral artery diseases, cardiovascular diseases, cognitive impairment, diabetes, and obesity are known to correlate with a higher mortality of elderly patients affected by COVID-19 [12].

## 5. Conclusions

Our study revealed greater fatality rates in males, indicating that sex may be a factor in severe COVID-19 consequences, such as mortality. Our results underscored the significance of implementing sex-specific preventive or treatment strategies for COVID-19 patients. The demographics of the population should be considered while making treatment decisions, with a focus on the prevalence of those 65 and older in the US population. Furthermore, monitoring the interactions and dynamics of the older population may offer additional suggestions to safeguard the more vulnerable older population. Sex-disaggregated data on COVID-19 infection and case fatality rates are necessary to develop gender-inclusive responses during the pandemic by scholars and policy experts. Sex-disaggregated data is required for the development of gender-equitable solutions for the pandemic. High-quality sex-disaggregated data should cover the population subgroups of all socioeconomic strata. Understanding other social factors linked to COVID-19 mortality will enable the identification of the most vulnerable populations in this pandemic, which will ensure an improved targeting, prevention, and intervention efforts. Assessing sex-disaggregated data is needed to address numerous knowledge gaps such as the immune system, comorbidities, the presumed infection source, and individual behaviors. Such information can contribute to the development of evidence-based guidelines for decision-making aimed at reducing these inequalities in the healthcare system.

## Use of AI tools declaration

The authors declare they have not used Artificial Intelligence (AI) tools in the creation of this article.
